# The Recombinant Bacille Calmette–Guérin Vaccine VPM1002: Ready for Clinical Efficacy Testing

**DOI:** 10.3389/fimmu.2017.01147

**Published:** 2017-09-19

**Authors:** Natalie E. Nieuwenhuizen, Prasad S. Kulkarni, Umesh Shaligram, Mark F. Cotton, Cyrill A. Rentsch, Bernd Eisele, Leander Grode, Stefan H. E. Kaufmann

**Affiliations:** ^1^Department of Immunology, Max Planck Institute for Infection Biology, Berlin, Germany; ^2^Serum Institute of India Pvt. Ltd., Pune, India; ^3^Stellenbosch University, Tygerberg, South Africa; ^4^Department of Urology, University Hospital Basel, Basel, Switzerland; ^5^Swiss Group for Clinical Cancer Research (SAKK), Bern, Switzerland; ^6^Vakzine Projekt Management GmbH, Hannover, Germany

**Keywords:** tuberculosis, bacille Calmette–Guérin, VPM1002, vaccine, listeriolysin, immune response

## Abstract

The only licensed vaccine against tuberculosis (TB), bacille Calmette–Guérin (BCG), protects against severe extrapulmonary forms of TB but is virtually ineffective against the most prevalent form of the disease, pulmonary TB. BCG was genetically modified at the Max Planck Institute for Infection Biology to improve its immunogenicity by replacing the urease C encoding gene with the listeriolysin encoding gene from *Listeria monocytogenes*. Listeriolysin perturbates the phagosomal membrane at acidic pH. Urease C is involved in neutralization of the phagosome harboring BCG. Its depletion allows for rapid phagosome acidification and promotes phagolysosome fusion. As a result, BCGΔ*ureC*::*hly* (VPM1002) promotes apoptosis and autophagy and facilitates release of mycobacterial antigens into the cytosol. In preclinical studies, VPM1002 has been far more efficacious and safer than BCG. The vaccine was licensed to Vakzine Projekt Management and later sublicensed to the Serum Institute of India Pvt. Ltd., the largest vaccine producer in the world. The vaccine has passed phase I clinical trials in Germany and South Africa, demonstrating its safety and immunogenicity in young adults. It was also successfully tested in a phase IIa randomized clinical trial in healthy South African newborns and is currently undergoing a phase IIb study in HIV exposed and unexposed newborns. A phase II/III clinical trial will commence in India in 2017 to assess efficacy against recurrence of TB. The target indications for VPM1002 are newborn immunization to prevent TB as well as post-exposure immunization in adults to prevent TB recurrence. In addition, a Phase I trial in non-muscle invasive bladder cancer patients has been completed, and phase II trials are ongoing. This review describes the development of VPM1002 from the drawing board to its clinical assessment.

## Introduction

Infection with *Mycobacterium tuberculosis* (*Mtb*) led to 10.4 million recorded cases of tuberculosis (TB) in 2015, with 1.8 million recorded deaths [World Health Organization (WHO) report 2016]. The current therapy involves 6–9 months of antibiotics, with the emergence of multiple drug resistant strains being a continuing obstacle. An attenuated form of the bovine *Mycobacterium* species, *Mycobacterium bovis* bacille Calmette–Guerin (BCG) has been in clinical use since 1921 and remains the only licensed vaccine against TB. BCG partially protects against TB meningitis and disseminated TB in infants and has non-specific immunostimulatory effects (1), which reduce general infant mortality by enhancing responses to other infectious diseases (2, 3). However, in all age groups, BCG does not adequately protect against pulmonary TB, the most prevalent form of disease and the route of disease transmission. In addition, BCG can cause severe adverse effects in immunocompromised individuals (4) and hence is contraindicated in HIV-infected individuals, the group that is most vulnerable to TB. However, in the absence of an alternative, BCG continues to be used in the immunization programs of several countries. To overcome these issues, several TB vaccine candidates are under development (5). One of the most advanced among them is BCG *ΔureC*::*hly* (VPM1002) (6).

**Figure 1:**
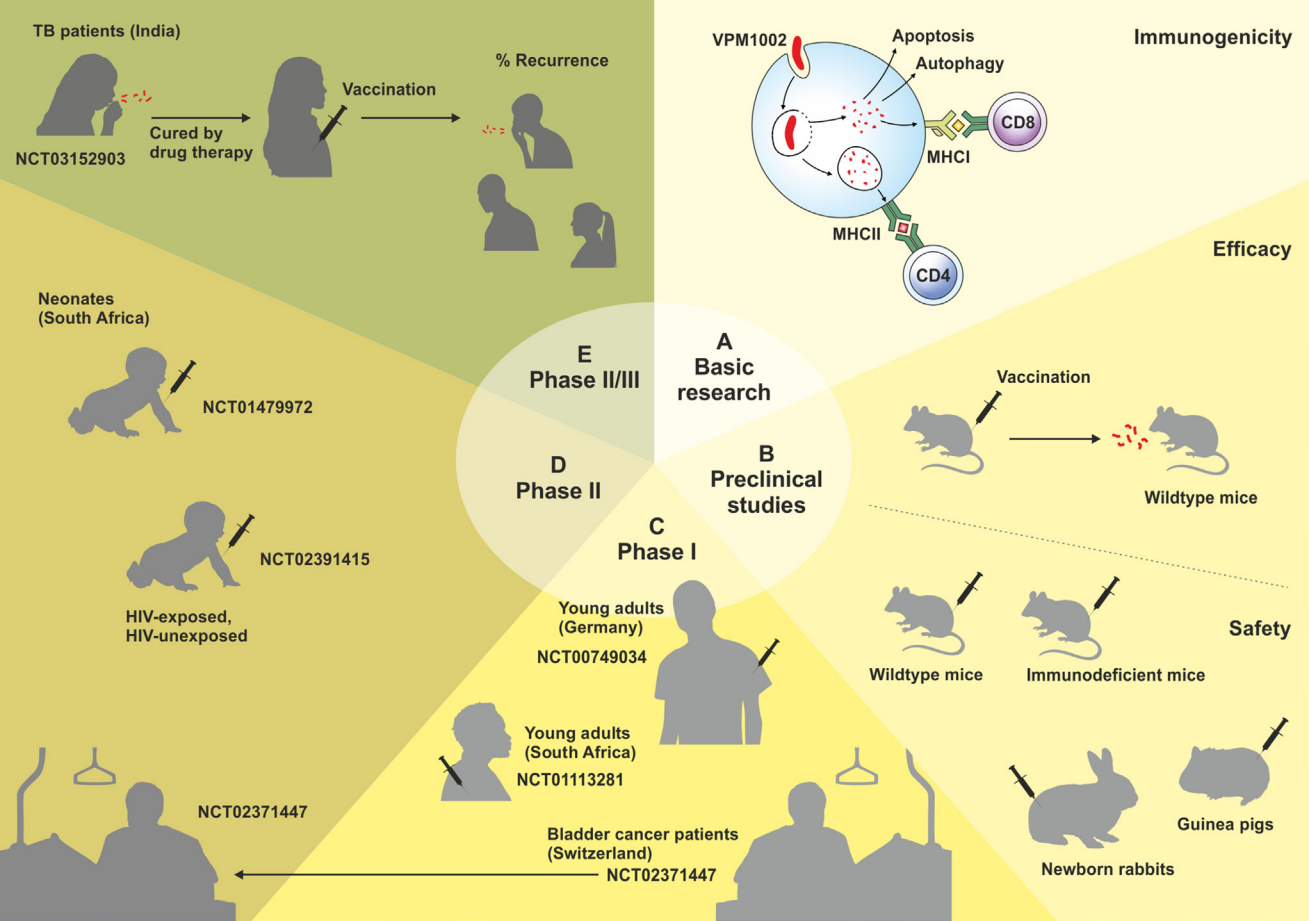
Schematic overview of the development of the VPM1002 vaccine candidate. Clinical trials are labeled by their ClinicalTrials.gov Identifier number.

VPM1002 is a recombinant BCG (rBCG) in which the urease C gene has been replaced by the listeriolysin O (LLO) encoding gene (*hly*) from *Listeria monocytogenes* (7). Urease C drives neutralization of phagosomes containing mycobacteria by generation of ammonia, thereby inhibiting phagolysosomal maturation and contributing to the survival of mycobacteria inside the macrophage (8, 9). Its depletion allows for rapid phagosome acidification, which promotes phagolysosome fusion and provides the optimal pH for LLO stability (10). LLO is a cholesterol-dependant cytolysin that forms transmembrane β-barrel pores in the phagolysosome membrane, allowing escape of *L. monocytogenes* into the cytosol (10, 11). Its expression in VPM1002 results in the release of antigens and bacterial DNA into the cytosol, triggering autophagy, inflammasome activation, and apoptosis. VPM1002 has demonstrated substantially increased immunogenicity, efficacy, and safety in preclinical studies, successfully passed Phase I and II clinical trials, and will now enter a Phase II/III clinical trial in India in 2017. This review summarizes the development, preclinical, and clinical testing of VPM1002 (Figure 1).

## Design and Generation of VPM1002

The attenuation of BCG was achieved by passaging virulent *M. bovis* in bile-containing medium for 13 years in the laboratory (12), during which time several genome segments were lost, including a segment known as Region of Difference 1 (RD1) which encodes the unique mycobacterial ESX-1 type VII secretion system (13, 14). ESX-1-dependent perturbation of host cell membranes requires direct contact with pathogenic mycobacteria such as *Mtb*, allowing the bacilli or their antigens to egress the phagosome into the cytosol (15). *Mtb* antigens are thus accessible to both the endocytic major histocompatibility complex (MHC) class II antigen presentation pathway and the MHC I antigen presentation pathway in the cytosol, and consequently can stimulate CD4^+^ and CD8^+^ T-cell subsets, respectively, both of which are required for optimal protection against TB (16–21). In addition, ESX-1 dependent release of *Mtb* DNA into the cytosol can be detected by host sensors, leading to activation of NLR family pyrin domain-containing 3 (NLRP3) and absent in melanoma 2 inflammasomes, release of interferons, increased autophagy and apoptosis (22–25). Induction of apoptosis in infected host cells generates vesicles carrying mycobacterial antigens that can be phagocytosed by bystander antigen presenting cells, mainly dendritic cells (DCs) and trafficked through MHC I antigen processing pathways to stimulate CD8^+^ T cells in a process known as cross-priming (26, 27). Mice with deficient cross-presentation due to the absence of annexin 1 show impaired *Mtb*-specific CD8^+^ T cells and are highly susceptible to TB (28). Lacking the ESX-1 secretion system, BCG is restricted to the phagosome of host cells, therefore its antigens and bacterial DNA do not enter the cytosol and the antigens are primarily processed by MHC class II pathways, stimulating CD4^+^ T cell responses (13, 14, 29, 30). BCG induces only weak apoptosis and CD8^+^ T cell responses (26). Furthermore, both BCG and *Mtb* inhibit surface MHC II expression, as urease-dependent alkalinization of the phagosome causes intracellular sequestration of MHC II dimers, resulting in suboptimal CD4^+^ T cell responses (31–33). Phagosomal biology is therefore a clear target for interventions aimed at enhancing T cell responses against mycobacteria.

Originally, VPM1002 was designed to improve accessibility of mycobacterial antigens to the MHC I pathway via cytosolic egression of antigens mediated by LLO perturbation of phagosomal membranes in order to improve induction of CD8^+^ T cells by the parental BCG strain (34, 35). In addition, leakage of phagolysosomal proteases such as cathepsins into the cytosol could activate caspases, leading to apoptosis and subsequent cross-presentation of mycobacterial antigens, which promotes both MHC I and MHC II restricted T cell stimulation (36). Studies with *L. monocytogenes* have shown that pore formation by LLO also triggers many downstream effects such as activation of the NLRP3 inflammasome, induction of cytokine expression, activation of kinases, triggering of endocytosis, histone modification and release of calcium from intracellular stores (37). An Hly recombinant strain, *hly*^+^ rBCG^+^, was generated by integrating the *hly* gene into BCG using the mycobacteria-*Escherichia coli* shuttle vector pMV306 (34). LLO was detected in the membrane structures, phagosomal space, and cytoplasmic vacuoles of macrophages infected with BCG pMV306::*hly*, and intracellular persistence of this strain was reduced compared with the parental BCG strain. MHC I presentation of co-phagocytosed soluble protein was improved in macrophages infected with this strain compared to BCG (34) and an *in vitro* human cytotoxic T lymphocyte (CTL) assay using cultured DCs and T cells from healthy human donors demonstrated that *hly^+^* BCG infection was better at inducing CTL responses than BCG infection (38). In the next generation strain, deletion of *ureC* was performed to ensure an optimal (acidic) pH for LLO stability; however, absence of *ureC* also promotes MHCII trafficking to the macrophage surface (31), which would also stimulate CD4^+^ T cell responses. To generate Δ*ureC hly*^+^ BCG, the chromosomal integrative shuttle vector pMV306*hyg-hly* (8) was used to transform *M. bovis* BCG Δ*ureC*::*aph*, and hygromycin-resistant clones were selected (35). The vaccine was licensed to Vakzine Projekt Management, and named “VPM1002.” The resistance cassette was subsequently successfully removed, although VPM1002 is equally sensitive to the antimycobacterial agents isoniazid, rifampicin, and ethambutanol in the presence or absence of the hygromycin resistance gene (39).

## Host Cell Responses to VPM1002 *In Vitro*

Increased quantities of mycobacterial antigen were detected in VMP1002 infected macrophages compared to BCG infected macrophages (35), and mycobacterial DNA was detected only in the cytosol of VPM1002 infected but not BCG infected macrophages (29), indicating that expression of LLO in BCG Δ*ureC*::*hly* allows the escape of bacterial products to the cytosol, presumably by perturbation of the phagosomal membrane. The bacteria themselves do not escape to the cytosol, unlike *Mtb* bacilli (29, 35). Infection of primary human and mouse macrophages demonstrated increased apoptosis after infection with VPM1002 compared to both BCG and BCG::*hly*, demonstrating the additional benefit of urease C deletion (35). Membrane disruption can facilitate the release of phagolysosomal proteases such as cathepsins into the cytosol, which are known to induce apoptosis (36, 40). Both the presence of mycobacterial proteins in the cytosol and the induction of apoptosis by perforation of the phagosomal membrane could cause increased trafficking of antigens to MHC I pathways (35). Apoptosis results in an increase in both CD8^+^ and CD4^+^ T cell responses in mycobacterial infection, suggesting that DCs may transfer efferocytosed antigens to the endocytic system (27, 36). The priming potential of apoptotic vesicles isolated from BCG and VPM1002 infected mouse macrophages was investigated in a co-culture system with splenic DCs and T cells, and VPM1002-infected apoptotic vesicles induced more profound CD4^+^ and CD8^+^ T cell responses compared to those infected with BCG (41). Vesicles from VPM1002 infected macrophages also induced higher production of the T helper type (Th)17-polarizing cytokines interleukin (IL)-6 and IL-23, and the immunoregulatory cytokine IL-10 by bone marrow-derived DCs.

Experiments in THP1 macrophages demonstrated that VPM1002 infection leads to activation of multiple caspases (29). The apoptotic effector caspases 3 and 7 were highly activated by VPM1002 in comparison to BCG, as well as caspase 1, which mediates pyroptosis, an inflammatory form of cell death and is an important regulator of the inflammatory response (42). Inflammasomes are multi-protein complexes composed of intracellular sensors and caspase 1. They control activation of caspase 1, which in turn cleaves the precursors of the cytokines IL-1β and IL-18 into their active forms (43). VPM1002 infection increased production of IL-1β and IL-18, which was dependent on AIM2 inflammasome activation but not on NLRP 1 and 3 inflammasome activation. Furthermore, VPM1002 induced increased levels of the autophagy marker microtubule-associated protein light chain 3 in an AIM2- and stimulator of interferon genes (STING)-dependent manner. The AIM2 inflammasome senses cytosolic DNA and is involved in the induction of caspase 1-dependent pyroptosis (44, 45), while STING acts as an essential adaptor protein in the induction of autophagy by cytosolic DNA (25). Autophagy, a protein degradation process induced by stress conditions such as infection, promotes the delivery of cytosolic antigens to MHC trafficking pathways (46, 47). It has also been shown to contribute to innate immunity against mycobacteria and other intracellular pathogens (48, 49). While autophagy was originally thought to be non-specific, it is now known that it can selectively target intracellular pathogens in a process known as xenophagy that involves ubiquitination of pathogen proteins or pathogen-containing endosomes (50). Intriguingly, gene expression of guanylate-binding proteins (GBPs) was also elevated in VPM1002 infected THP-1 macrophages compared to BCG infected macrophages. Interferon-inducible GBPs have multiple roles in inflammasome activation, autophagy, and lysis of pathogen-containing vacuoles and can even directly target the pathogens themselves (51–54). Whether they play a role in the translocation of mycobacterial components from the phagosome into the cytosol during VMP1002 infection remains to be determined.

Disruption of the VPM1002-containing phagosome membrane by LLO and release of mycobacterial DNA into the cytosol appears to have effects in inducing immune responses that are similar to the effects of ESX-1 activity in *Mtb* or *M. marinum*. ESX-1 of *M. marinum* stimulates autophagosome formation and recruitment to the vacuole; however, unlike LLO it also inhibits autophagic flux, thereby preventing bacterial degradation (49). Testing of vaccine candidates expressing ESX-1 such as *Mtb* Δ*ppe25-pe19* (55) and BCG expressing ESX-1 of *M. marinum* (BCG:ESX-1^Mmar^) (56) demonstrated that ESX-1 was critical for enhancing innate immune responses via phagosome rupture. BCG:ESX-1^Mmar^ induced the cGas/STING/TBK1/IRF-3/type I interferon axis and promoted AIM2 and NLRP3 inflammasome activation, resulting in increased frequencies of antigen-specific CD8^+^ and CD4^+^ T cells and increased protection against *Mtb* compared to BCG (56), while *Mtb* Δ*ppe25-pe19* also led to enhanced protection. ESX-1 may induce protective immunity by an additional mechanism, as ESAT6 is required for rapid, non-cognate IFN-γ production by CD8^+^ T cells, mediated by the NLRP3/caspase-1/IL-18 axis (57).

## Preclinical Efficacy and Safety

Aerosol challenge of vaccinated BALB/c or C57BL6 mice with 100–200 colony-forming units (CFUs) of *Mtb* H37Rv or a clinical isolate of the Beijing/W genotype family demonstrated that VPM1002 immunization has significantly greater protective efficacy than the parental BCG strain, with bacterial loads in the lungs typically reduced by one to two logs in late stages of infection (35, 58–61). In a low dose infection (30 CFU), VPM1002 led to an almost 1000-fold reduction of *Mtb* in the lungs compared to naïve mice at day 200 after infection (35). Homologous boosting with VPM1002 did not improve protection compared to a single immunization (60). However, a post-exposure vaccination model using antibiotics for an extended period and then allowing bacterial regrowth demonstrated that mice with subclinical TB had lower bacterial burdens when vaccinated with VPM1002 compared to BCG, suggesting that VPM1002 could also be considered for use as a post-exposure vaccine (60).

The safety profile of VPM1002 has been evaluated in animal models including mice, guinea pigs, rabbits, and non-human primates (6). In *RAG1-/-* immunodeficient mice lacking mature T and B cells, bacterial loads were not significantly different in lungs and spleen after vaccination with VPM1002 compared to BCG (35). However, VPM1002 demonstrated substantially lower virulence in severe combined immunodeficiency mice, most likely due to the reduced intracellular persistence of this strain (35, 61). After immunization of wildtype BALB/c or C57BL6 mice, VPM1002 was more rapidly cleared from the draining lymph nodes than BCG and disseminated less to the spleens, where it was also quickly cleared (59, 61). Dissemination to the lungs was observed in BCG vaccinated but not VPM1002 vaccinated mice. Enhanced adaptive immune responses after VPM1002 vaccination are therefore likely to play a role in the reduced dissemination of VPM1002 in immunocompetent mice. Overall, the data demonstrate increased safety and protective efficacy of VPM1002 compared to parental BCG in mice.

In guinea pigs and non-human primates, the safety of VPM1002 was comparable to that of BCG (6, 39). As the primary target population for vaccination against TB is newborns, the safety profiles of VPM1002 and BCG were also compared in newborn rabbits (39). No dissemination to tissues was observed after VPM1002 administration, and the body weight gain was not affected during the 90 days observation period, whereas the body weight was reduced in the BCG vaccinated group compared to the saline control group. No premature mortality was observed in either group. The preclinical safety of VPM1002 is thus supported by a large body of evidence.

## Analysis of Immune Responses to VPM1002 in Mice

Analysis of gene expression in mice early after immunization with VPM1002 demonstrated that, as in THP-1 cells, expression of IL-18 and IL-1β was increased, as well as expression of IFN-inducible genes such as *Tmem173* (STING), *Gbp*’s, and other GTPases (29, 61). Apoptosis was increased in the lymph nodes of VPM1002 immunized mice compared with BCG immunized mice by day 14 (61). Immunization with VMP1002 induced both type 1 and type 17 cytokine responses in mice, whereas BCG induced type 1 responses only (58). After restimulation with PPD, levels of IFN-γ, IL-17, IL-2, IL-6, and GM-CSF were increased in lung cells isolated from VPM1002 immunized mice compared to those from BCG immunized mice, and splenocytes from VPM1002-vaccinated mice also produced more IL-17. Furthermore, percentages of γδ T cells producing IFN-γ and IL-17 were increased after vaccination with VPM1002 (58). Seven days after *Mtb* challenge, IL-2^+^TNF^+^ double cytokine producing cells were increased in the lungs of VPM1002-immunized mice compared with BCG-vaccinated mice, suggesting recall responses, because newly generated T cells take 12–14 days to reach the lungs during *Mtb* infection (62). IL-2^+^TNF^+^ CD4^+^ T cells typically show a central memory phenotype (T_CM_) (21), and further studies demonstrated that VPM1002 immunization indeed induces higher frequencies of T_CM_ than immunization with BCG (59, 61). Ag85B-specific CD4^+^ T_CM_ were significantly increased in the draining lymph nodes of VPM1002-vaccinated compared to BCG-vaccinated mice at day 14 (59).

Bacille Calmette–Guérin induces effector memory CD4^+^ T (T_EM_) cells that can control acute infection but appears to induce insufficient numbers of T_CM_ cells for long-term protection (21). Transfer studies demonstrated that T_CM_ cells from VPM1002 infected mice conferred protection against TB infection whereas T_EM_, T follicular helper (T_FH_), and naïve T cells did not, at least at the numbers of cells tested (59). These findings concur with other studies in which T_CM_ cells were associated with protection (21). While T_EM_ cells appear early after infection and provide protection by the secretion of effector cytokines such as IFN-γ and TNF-α, T_CM_ cells proliferate in the LN and generate new pools of T_EM_ cells after re-exposure to antigen (59, 63, 64). The T_CM_ cells generated by subcutaneous vaccination with VPM1002 or BCG were found to reside over the long term in the secondary lymphoid organs, rather than in the lung, and to be recruited to the lungs after *Mtb* challenge (58, 59). Waning of BCG-induced immunity correlates with a decline in T cell functions such as cytokine production and CTL activity and an increase in terminally differentiated, dysfunctional T cells (65). Thus, systemic maintenance of T_CM_ populations over the long term and the rapid recruitment of T_CM_ cells to the lung following *Mtb* infection remains a key goal in the development of more effective vaccine candidates (59). VPM1002 also induced an increase in mycobacteria-specific immunoglobulin G levels after vaccination compared to BCG, and a concomitant increase in CXCR5-expressing T_FH_ cells (59, 61), which have been associated with decreased lung pathology (66) and stimulate germinal center B cell responses (63). Passive transfer of serum from VPM1002- or BCG-immunized mice on the day of *Mtb* infection and thrice weekly did not reduce bacterial load at day 14 (59), but growing evidence suggests that antibodies may play a role in protection against *Mtb* (67–71). Overall, increased protection conferred by VPM1002 immunization in the mouse model was associated with increased numbers of T_CM_ and T_FH_ cells, increased Th17 responses, earlier recruitment of T cells to the lungs following *Mtb* challenge and increased levels of anti-mycobacterial antibodies (58, 59, 61).

## Clinical Trials with VPM1002: A Step Toward a Safer, More Efficacious TB Vaccine

Human data on VPM1002 are available from three clinical trials, all performed with the original hygromycin-resistant strain of VPM1002. Two Phase I studies were performed in healthy adult volunteers, and one Phase IIa study was conducted in healthy newborn infants, one of the intended target populations. In the first Phase I clinical trial (ClinicalTrials.gov Identifier: NCT00749034) conducted in Germany, healthy Caucasian adult males with (W) or without (WO) a history of BCG vaccination received VPM1002 randomized to three escalating doses (*N* = 30W + 30WO) or BCG at the standard vaccine dose (*N* = 10W + 10WO) and were followed for 6 months. Single vaccination with VPM1002 up to 5 × 10e5 CFU was safe and well tolerated. The immunogenicity of VPM1002 as measured by IFN-γ release by stimulated T cells was dose dependent. Both VPM1002 and BCG induced multifunctional CD4^+^ and CD8^+^ T cell subsets, which are thought to play a role in protection against TB (72–74), with VPM1002 showing an earlier increase in double and triple cytokine producing T cells which remained at heightened levels throughout the study (7). Furthermore, only VPM1002 induced serum antibodies against mycobacterial antigens (7), echoing preclinical studies in which VPM1002 induced higher levels of mycobacteria-specific antibodies than BCG in mice (59, 61). In the second Phase I clinical trial (ClinicalTrials.gov Identifier: NCT01113281), performed in South Africa, 24 healthy male and female adults with a history of BCG immunization, predominantly from the indigenous African population, were vaccinated with VPM1002. The study showed that a single vaccination with VPM1002 is safe, well tolerated and elicits a profound immune response in an African adult population (6).

The Phase IIa clinical trial (ClinicalTrials.gov Identifier: NCT01479972) was the first investigation of VPM1002 in newborns (75). It was conducted in Cape Town, South Africa, a region with a high TB burden. Forty-eight HIV-unexposed, newborn infants were vaccinated with either VPM1002 (*n* = 36) or BCG (*n* = 12) through an open label, randomized, controlled design. Polyfunctional CD4^+^ and CD8^+^ T cell responses were similar between the groups, and both groups had increased IFN-γ responses after 7 h PPD stimulation at all measured time points post vaccination compared to baseline. Both vaccines induced IL-17 responses; though, unlike BCG, VPM1002 induced increased proportions of CD8^+^ IL-17^+^ T cells at day 14 and month 6 time points compared to the baseline. The incidence of abscess formation was lower for VPM1002 compared to BCG. Thus, VPM1002 was safe, well tolerated, and immunogenic in newborn infants.

In addition, a Phase IIb clinical trial is currently ongoing in South Africa (ClinicalTrials.gov Identifier: NCT02391415). This trial is a double-blind, randomized, controlled study to evaluate the safety and immunogenicity of VPM1002 in comparison with BCG in HIV-exposed uninfected (HEU) and HIV-unexposed, BCG-naive newborn infants. The inclusion of HEU infants in the trial is important, as this group comprises 30% of the newborns requiring BCG vaccination in South Africa, and they may be at higher risk of *Mtb* infection than HIV-unexposed infants. The proportion of HEU may vary in different countries. Previous work from Brazil suggests that HEU infants have poorer T-cell proliferation and lower levels of IFN-γ production compared to HIV-unexposed infants (76). Enrollment of 416 infants has been completed and follow-up is in progress. Follow-up will continue for 12 months, as opposed to 6 months in NCT01479972, enabling collection of preliminary efficacy data.

In addition to its development as a vaccine for newborns, VPM1002 is also being assessed as a post-exposure vaccine for adults, since preclinical studies in mice demonstrated that it reduced bacterial loads in a post exposure model (60). A phase II/III trial has received regulatory approval by the Indian authorities. Once ethics committee approvals are received for all sites, the trial will commence across India (ClinicalTrials.gov Identifier: NCT03152903). The study will be conducted in 2000 adults who were TB patients, but received drug treatment and were cured of disease. In such populations, there is a high risk of recurrence (including re-infection and relapse), especially within 12 months after completing treatment. The multi-centric, placebo-controlled, randomized, controlled study will assess whether VPM1002 can prevent such TB recurrence over a 1-year follow-up period. Currently, no intervention is licensed for this indication, including BCG, which means there is clearly an unmet medical need. The study will also expand the safety database on VPM1002.

## Evaluation of VPM1002 as a Bladder Cancer Therapy

Bladder cancer is the ninth most common cancer in the world, and is four times more common in men than in women (77). The main risk factors for developing bladder cancer include smoking, *Schistosoma* infection (bilharzia), and exposure to industrial chemicals (77, 78). Tumors can be non-muscle invasive, i.e., confined to the mucosa of the bladder wall, or muscle-invasive. More than seventy percent of bladder cancers are detected while they are still non-muscle invasive (79). Due to its immunostimulatory properties, repeated intravesical BCG instillation is the standard adjuvant treatment for intermediate to high-risk non-muscle-invasive bladder cancer (NMIBC) after transurethral resection of the tumors (80–82). BCG therapy reduces the risk of recurrence and the progression to muscle invasive bladder cancer. The repeated instillations require much higher doses and volumes of BCG than vaccination against TB does, and some patients have adverse events that lead to discontinuation of the therapy (83, 84). Adverse events include fever, bladder irritation, decreased bladder capacity, incontinence, hematuria, flu-like symptoms and in approximately 5% of cases, BCG infection (85, 86). Patients undergoing traumatic catheterization are at risk for intraluminal BCG dissemination, resulting in a potentially lethal systemic infection (87).

The precise immune mechanisms by which BCG promotes anti-tumor activity in bladder cancer are not completely resolved, but it is well-established that the ability of BCG to promote Th1 responses is important, as well as the recruitment of neutrophils and innate lymphocytes including natural killer cells (82, 88, 89). Activation of immune cells may lead to elimination of the urothelial cancerous cells that have internalized BCG (82, 90). Increased CD4^+^ T cell responses have been measured during BCG therapy, and BCG was shown to promote secretion of both Th1- and Th2-type cytokines (88, 91–93). A positive response to BCG therapy (no recurrence or evidence of disease during follow-up examinations) has been associated with an intratumoral Th2 predisposition (increased GATA3) and decreased concentrations of IL-10, combined with a Th1 functional phenotype indicated by increased levels of Th1-related inflammatory metabolites (88). In another study, increased regulatory T cells and tumor-associated macrophages in the tumor microenvironment were also associated with non-responsiveness, while increased GATA3^+^ and CD4^+^ T cells were associated with responders (88, 94). BCG Connaught conferred greater 5-year recurrence-free survival than BCG Tice and induced stronger Th1 type responses, BCG-specific CD8^+^ T cells and T cell recruitment to the bladder (93). Genetic analysis demonstrated several differences between the two strains, including the absence of RD15 in BCG Connaught (93).

Because approximately 30–40% of patients do not respond to BCG therapy and others suffer from adverse events, rBCG technology has been tested for improving the efficacy and tolerability of BCG in bladder cancer therapy (82). rBCGs that have been modified to express immunostimulatory molecules, cytokines, or antigens have been tested in mice for their capacity to induce stronger and more specific immune responses. VPM1002 is currently being evaluated in SAKK 06/14, a Phase I/II trial for immunotherapy in patients with NMIBC (ClinicalTrials.gov Identifier: NCT02371447). The phase I part of the trial has been completed in Switzerland. Intravesical application of VPM1002BC demonstrated that the product is safe and well tolerated in NMIBC patients. The recommended phase II dose has been established as 1–19.2 × 10e8 CFUs of VPM1002BC. The phase II part has been approved by the Swiss and German regulatory authorities and is currently ongoing in both countries.

## Outlook

The available preclinical and clinical data reveal that VPM1002 is immunogenic and may be better than BCG in terms of safety. VPM1002 could be a safe, well-tolerated and efficacious alternative to the BCG vaccine in the future. With an annual capacity of 100 million doses, Serum Institute of India Pvt. Ltd. can meet the global demand for a BCG vaccine and is well poised to supply the new vaccine if efficacy trials are successful. While this vaccine progresses through efficacy trials, next-generation derivatives are being designed and tested in preclinical models aimed at optimizing efficacy and/or safety (61, 95). Furthermore, VPM1002 is currently being tested in goats by the Friedrich Loeffler Institute in Germany for the prevention of *M. caprae* infection (Menge et al. unpublished data). Infections with *M. caprae* and *M. bovis*, closely related species of the same clade that cause TB in goats and cattle, respectively, are of agricultural importance, and can potentially be transmitted to humans (96, 97).

Almost 100 years after the first immunization with BCG, a rBCG vaccine candidate is ready for clinical efficacy testing. This marks a major step forward in the long journey that began when the recombinant vaccine was constructed in the late 1990s and tested in different animal models to determine its safety and protective effect.

## Author Contributions

NN, LG, and SK wrote and reviewed the manuscript. All other authors (BE, PK, US, CR, and MC) reviewed the manuscript.

## Conflict of Interest Statement

SK and LG are co-inventors/patent holders of BCG Δ*ureC*::*hly* (VPM1002). BE and LG are working for the Vakzine Projekt Management GmbH who is involved with the development of VPM1002. PK and US are employed by Serum Institute of India Pvt. Ltd., which manufactures VPM1002. NN, CR, and MC declare that they have no conflicts of interest.

## Abbreviations

AIM2, absent in melanoma 2; BCG, bacille Calmette–Guérin; CFUs, colony forming units; CTL, human cytotoxic lymphocyte; DC, dendritic cell; EPI, expanded program of immunization; GBP, guanylate binding protein; HEU, HIV-exposed uninfected; IgG, immunoglobulin G; LC3, microtubule-associated protein light chain 3; LLO, listeriolysin O; MHC, major histocompatibility complex; *Mtb, Mycobacterium tuberculosis*; NLRP3, NLR family pyrin domain-containing 3; NMIBC, non-muscle-invasive bladder cancer; rBCG, recombinant BCG; RD1, Region of Difference 1; SCID, severe combined immunodeficiency; STING, stimulator of interferon genes; TB, tuberculosis; T_CM_, central memory T cells; T_EM_, effector memory T cells; T_FH_, follicular T cells; Th, T helper cell.
